# Inclusion of genetically identical animals to a numerator relationship matrix and modification of its inverse

**DOI:** 10.1186/1297-9686-41-25

**Published:** 2009-03-03

**Authors:** Takuro Oikawa, Kazuhiro Yasuda

**Affiliations:** 1 Graduate School of Natural Science and Technology, Okayama University, Okayama-shi 700-8530, Japan

## Abstract

In the field of animal breeding, estimation of genetic parameters and prediction of breeding values are routinely conducted by analyzing quantitative traits. Using an animal model and including the direct inverse of a numerator relationship matrix (NRM) into a mixed model has made these analyses possible. However, a method including a genetically identical animal (GIA) in NRM if genetic relationships between pairs of GIAs are not perfect, is still lacking. Here, we describe a method to incorporate GIAs into NRM using a **K **matrix in which diagonal elements are set to 1.0, off-diagonal elements between pairs of GIAs to (1-x) and the other elements to 0, where x is a constant less than 0.05. The inverse of the **K **matrix is then calculated directly by a simple formula. Thus, the inverse of the NRM is calculated by the products of the lower triangular matrix that identifies the parents of each individual, its transpose matrix, the inverse of the **K **matrix and the inverse of diagonal matrix **D**, in which the diagonal elements comprise a number of known parents and their inbreeding coefficients. The computing method is adaptable to the analysis of a data set including pairs of GIAs with imperfect relationships.

## Introduction

Cloning animals is regarded as a means to multiply genetically identical animals (GIAs). In Japan, clones of bulls are routinely produced to test bulls' performance and in some cases to multiply fattening animals. A survey conducted by the Japanese Ministry of Agriculture, Forestry and Fisheries, and published on October 31, 2007, has recorded calves cloned from somatic cells of 535 animals and from embryonic cell nuclei of 716 animals.

In animal breeding, analysis of quantitative traits using a mixed model is essential to predict the breeding value of an individual and to estimate the genetic parameters of the traits. When applying an animal model to perform the genetic analysis, it is necessary to include the inverse of the numerator relationship matrix (NRM) in order to connect all the animals included in the mixed model; however, calculating the inverse of a large NRM requires exceptionally large computing power. On the one hand, Henderson [1] has developed a method of calculating directly **A**^-1^, without calculating the **A **matrix itself in a non-inbred population. This innovation has made it possible to use a model in which the data set includes a large number of animals. On the other hand, Quaas [2] has extended the method for the application to inbred populations by including the inbreeding coefficients in the model. A faster computing method of inbreeding coefficients has been developed by Tier [3] and Meuwissen and Luo [4], where inbreeding coefficients are computed as a subset of the **A **matrix. In addidion, Famula [5] has proposed a simplified algorithm for inbred populations, incorporating parental uncertainty to the model.

Inclusion of GIAs in the model raises the problem of a singular **A **matrix because of perfect additive genetic relationships between pairs of GIAs. In the case of an analysis with a singular **A**, Henderson [6] presented a method to solve a mixed model without inversion of the **G **matrix, where **G **= **A**$\sigma_{a}^{2}$. A few years later, Kennedy and Schaeffer [7] proposed a model in which records on GIAs are treated as repeated records on the same genotype. Their model assumes perfect genetic relationships among GIAs. However in reality, the genetic relationship between pairs of GIAs is not perfect because genetic diversity between such pairs originates from several genetic factors such as the difference in the recipient cytoplasm, mutations in the somatic cell and gene imprinting in the nucleus of the somatic cell [8-10]. Recently a study on human monozygotic twins analysing copy number variation, has revealed that genetic and phenotypic diversities exist even in monozygotic twins within pairs [11].

The objective of this study was to develop a method to compute directly the inverse of the NRM that includes GIAs with imperfect additive genetic relationships within pairs of GIAs.

## Methods

### Procedure

The **A **matrix is decomposed according to Famula [5].

(1) A=(I−12P)−1D(I−12P′)−1,

where **I **is the identity matrix, **P **is a lower triangular matrix which identifies the parents of each individual in the population. The **D **matrix is a diagonal matrix with *d*_*i*_, *i*th diagonal element of **D**. Then

(2) di={12−14Fp−14Fqif both parents of i, say p and q, are known,34−14Fpif only one parent, say p, is known,1if neither parent is known,

where *F*_*p *_and *F*_*q *_are the inbreeding coefficients of the parents of the *i*th animal. In (1), (I−12P) is the lower triangular matrix, and (I−12P′) is its transpose matrix. From(1), the inverse of the **A **matrix is as follows:

(3) A−1=(I−12P′)D−1(I−12P).

Next, we introduce the **K **matrix into (1) so that the **A **matrix includes GIA. Then, **A **is

(4) A≈(I−12P)−1DK(I−12P′)−1

and

(5) A−1≈(I−12P′)K−1D−1(I−12P),

where **K **is the matrix where a diagonal element is set to 1 and the off-diagonal element is set to (1 - *x*) when *i*th and *j*th animals are genetically identical and *x *is a constant near 0; **K **is as follows:

$$K=\left[\begin{array}{ccccc} 1 & 0 & \cdots & & \\ 0 & \ddots & & & \\ \vdots & & 1 & 1-x & \\ & & 1-x & 1 & \\ & & & & \ddots \end{array}\right].$$

Generally, for a matrix with GIAs where the diagonal elements are set to 1, and the off-diagonal elements are (1 - *x*), the inverse matrix for n GIAs is

$${\left[\begin{array}{cccc} 1 & 1-x & 1-x & \cdots \\ 1-x & 1 & 1-x & \cdots \\ 1-x & 1-x & 1 & \cdots \\ \vdots & \vdots & \vdots & \ddots \end{array}\right]}^{-1}=\left[\begin{array}{cccc} l_{n} & m_{n} & m_{n} & \cdots \\ m_{n} & l_{n} & m_{n} & \cdots \\ m_{n} & m_{n} & l_{n} & \cdots \\ \vdots & \vdots & \vdots & \ddots \end{array}\right],$$

where $l_{n}=\frac{1+\left(n-2\right)\left(1-x\right)}{x\left\{1+\left(n-1\right)\left(1-x\right)\right\}}$, $m_{n}=-\frac{1-x}{x\left\{1+\left(n-1\right)\left(1-x\right)\right\}}$

and $l_{n}+\left(n-1\right)m_{n}=\frac{1}{1+\left(n-1\right)\left(1-x\right)}$.

Thus, **K**^-1 ^is calculated, and the diagonal element of **D**^-1 ^is 1di. Therefore, **A**^-1 ^is calculated directly by the product of the matrices without computing the inverse of **A**.

### Algorithm for computation

Provided *d*_*i *_is calculated by the methods of Quaas [2] and Famula [5], **A**^-1 ^is calculated directly by the following steps:

i) If both parents of *i*, say *p *and *q *are known,

   and when *i *has no GIA,

      add 1di to element (*i*, *i*),

      add −12(1di) to elements (*p*,  *i*), (*i*,  *p*), (*q*,  *i*) and (*i*,  *q*),

      add 14(1di) to elements (*p*,  *p*), (*p*,  *q*), (*q*,  *p*) and (*q*,  *q*);

   when *i *has n GIAs (*g*_*ij*_, j = 1, 2, ... n),

         add ln(1di) to element (*i*, *i*),

      add −12{ln+(n−1)mn}(1di) to elements (*p*,  *i*), (*i*, * p*), (*q*, * i*) and (*i*, * q*),

      add 14{ln+(n−1)mn}(1di) to elements (*p*, * p*), (*p*, * q*), (*q*, * p*) and (*q*,  *q*).

      If *i *is a donor animal of the GIAs,

         then add mn(1di) to elements (*i*, *g*_*ij*_) and (*g*_*ij*_, *i*).

ii) If only one parent, say p, is known,

   add 1di to element (*i*, *i*),

   add −12(1di) to elements (*p*, *i*) and (*i*, *p*),

   add 14(1di) to element (*p*, *p*).

iii) If neither parent is known,

   add 1 to element(*i*, *i*).

### Simulation

For the simulation study, animal phenotypes were generated assuming that the heritability of a trait was 0.5 and the variance for both additive genetic effect and random residuals was 2500. The number of animals in the base population (G0) was 300 (150 males and 150 females). Their phenotypic values were generated by the infinitesimal model using the random digits generator, ranlib [12]. The phenotypic value of a descendant animal in the latter two generations (G1 and G2) was formed by an average of the parents, a Mendelian sampling effect and a random residual. The number of animals was 750 (250 males and 500 females) in G1 and 1000 (no sex effect on recorded animals) in G2. The breeding animals in G1 were selected randomly. Records used to estimate variance components comprised only the phenotypic values of animals in G2. The number of GIAs for each mating was two, *i.e*., a total of 1000 GIAs (500 GIA pairs). The variance components were estimated by restricted maximum likelihood (REML) using remlf90 [13]. The number of replicates for the simulation was 20 for each x value (from 0.01 to 1.0).

## Results

### Effect of the K matrix

Let animal *j *be genetically identical to animal *i*. Unless their descendants at the *t*th generation are inbred animals with animal *i *or *j *as a common ancestor, the **K **matrix has the effect of adding *d*_*i*_(1 - *x*) to the NRM element of the GIA and of adding ${\left(\frac{1}{2}\right)}^{t-1}d_{i}\left(1-x\right)$ to the NRM element of their descendants at the *t*th generation. Thus, when there is no GIA, the off-diagonal element of animals *i *and *j *for the **A **matrix is *a*_*ij*_, and the off-diagonal element of animals *i *and descendant *s*_*t *_at the *t*th generation of animal *j *is $a_{is_{t}}$. If animals *i *and *j *have no common descendant, **A **is as follows:

$$A=\left[\begin{array}{ccccccccc} a_{11} & a_{12} & \cdots & & & & & & \\ & a_{22} & \cdots & & & & & & \\ & & \ddots & & & & & & \\ & & & a_{ii} & a_{ij}+d_{i}\left(1-x\right) & a_{ik_{1}}+\frac{1}{2}d_{i}\left(1-x\right) & a_{ik_{2}}+{\left(\frac{1}{2}\right)}^{2}d_{i}\left(1-x\right) & a_{ik_{3}}+{\left(\frac{1}{2}\right)}^{3}d_{i}\left(1-x\right) & \cdots \\ & & & & a_{jj} & a_{js_{1}} & a_{js_{2}} & a_{js_{3}} & \cdots \\ & & & & & a_{s_{1}s_{1}} & a_{s_{1}s_{2}} & a_{s_{1}s_{3}} & \cdots \\ & & & & & & a_{s_{2}s_{2}} & a_{s_{2}s_{3}} & \cdots \\ & & & & & & & a_{s_{3}s_{3}} & \cdots \\ & & & & & & & & \ddots \end{array}\right].$$

### Numerical example

**Figure 1:**
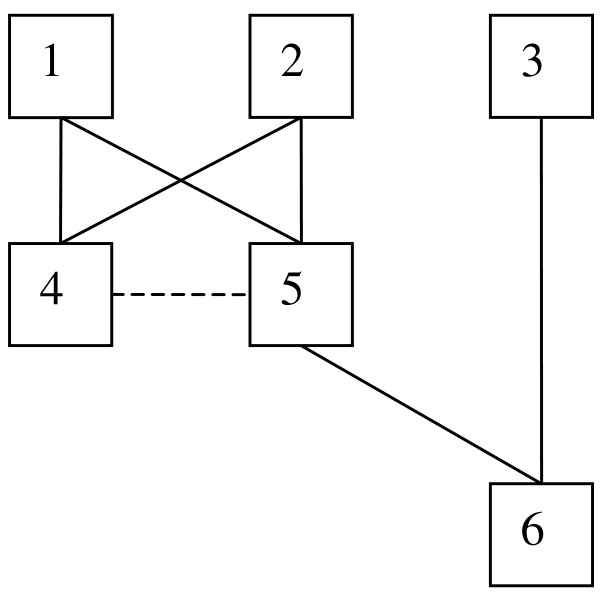
Diagram of the pedigree.

Our example uses a simple pedigree, where animals 1, 2 and 3 are in the base population. Animals 4 and 5 are the progeny of 1 and 2, and animal 6 is the progeny of 3 and 5. See Figure 1.

1) When animals 4 and 5 are full sibs,

the **P **matrix, which identifies the parents of the animals, is as follows:

$$P=\left[\begin{array}{cccccc} 0 & & & & & \\ 0 & 0 & & & 0 & \\ 0 & 0 & 0 & & & \\ 1 & 1 & 0 & 0 & & \\ 1 & 1 & 0 & 0 & 0 & \\ 0 & 0 & 1 & 0 & 1 & 0 \end{array}\right].$$

The **D **matrix has elements calculated in (2). Thus,

D=[11011201212].

Therefore, **A**^-1 ^in (3) is as follows:

A−1=(I−12P′)D−1(I−12P)=[100−12−12010−12−120100−1210001−121][11012022][1010001−12−1201−12−1200100−120−121]=[210−1−10120−1−100032012−1−1−10200−1−112052−100−10−12].

**A **from (4) is

$$A=\left[\begin{array}{cccccc} 1 & 0 & 0 & 1/2 & 1/2 & 1/4\\ & 1 & 0 & 1/2 & 1/2 & 1/4\\ & & 1 & 0 & 0 & 1/2\\ & & & 1 & 1/2 & 1/4\\ & & & & 1 & 1/2\\ & & & & & 1 \end{array}\right].$$

2) When animals 4 and 5 are GIAs,

the **K **matrix is as follows:

$$K=\left[\begin{array}{cccccc} 1 & 0 & 0 & 0 & 0 & 0\\ & 1 & 0 & 0 & 0 & 0\\ & & 1 & 0 & 0 & 0\\ & & & 1 & 1-x & 0\\ & & & & 1 & 0\\ & & & & & 1 \end{array}\right].$$

Then the inverse of the sub-matrix of **K **is

$${\left[\begin{array}{cc} 1 & 1-x\\ 1-x & 1 \end{array}\right]}^{-1}=\left[\begin{array}{cc} l_{2} & m_{2}\\ m_{2} & l_{2} \end{array}\right],$$

where $l_{2}=\frac{1+\left(2-2\right)\left(1-x\right)}{x\left\{1+\left(2-1\right)\left(1-x\right)\right\}}=\frac{1}{x\left(2-x\right)}$, $m_{2}=-\frac{1-x}{x\left\{1+\left(2-1\right)\left(1-x\right)\right\}}=-\frac{1-x}{x\left(2-x\right)}$, and $l_{2}+m_{2}=\frac{1}{1+(1-x)}$.

Therefore, **K**^-1 ^is

$$K^{-1}=\left[\begin{array}{cccccc} 1 & 0 & 0 & 0 & 0 & 0\\ & 1 & 0 & 0 & 0 & 0\\ & & 1 & 0 & 0 & 0\\ & & & \frac{1}{x\left(2-x\right)} & -\frac{1-x}{x\left(2-x\right)} & 0\\ & & & & \frac{1}{x\left(2-x\right)} & 0\\ & & & & & 1 \end{array}\right].$$

**A**^-1 ^in (5) is as follows:

A−1=[1+12−x12−x0−12−x−12−x01+12−x0−12−x−12−x032012−12x(2−x)−2(1−x)x(2−x)012+2x(2−x)−12]

Then **A **in (4) is

A=[1001212141012121410012112+12(1−x)14+14(1−x)1121].

Here, if *x *→ 0, **A **is as expected,

A=[100121214101212141001211121121].

Thus, the elements of animals 4 and 5 are those of the GIAs.

### Simulation

**Figure 2:**
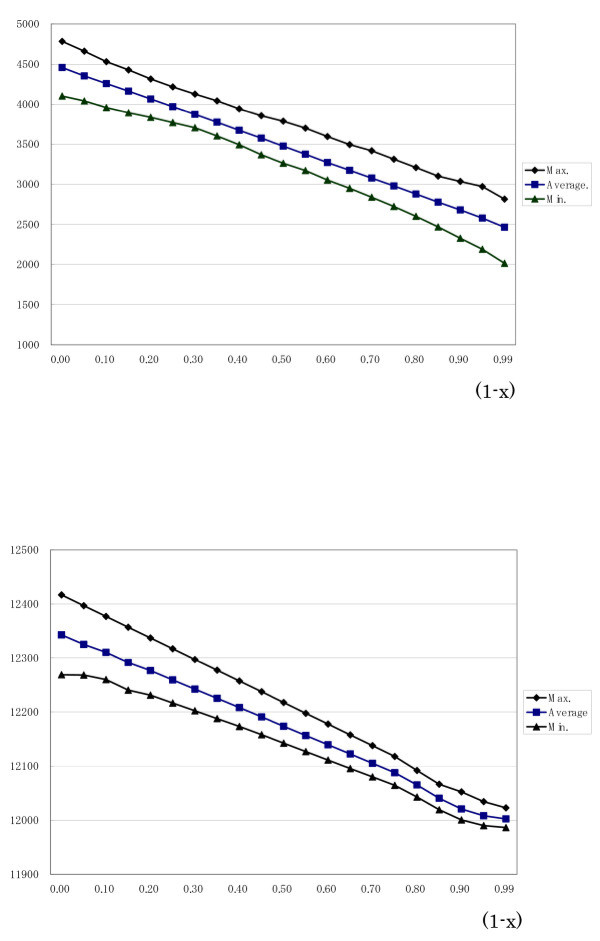
**a. Averages and ranges of estimated additive genetic variances for different (1-x) values with 20 simulated data sets**. b. Averages and ranges of log likelihood (-2logL) for different (1-x) values with 20 simulated data sets.

Figure 2a presents the results of the estimated genetic variance. Averages of 20 estimates are shown together with their ranges (minimum and maximum). Genetic variances were overestimated at low (1-x) and around the true value at high (1-x) and estimated residual variances were underestimated at low (1-x) and around the true value at high (1-x) (data not shown).

Figure 2b shows averages of log likelihood (-2logL) for various (1-x) values together with their ranges when REML estimates were obtained for the variance components. The log likelihood declined at high (1-x): 0.95 or 0.99, indicating the validity of the model with high (1-x). The difference between the models with 0.95 and 0.99 was statistically insignificant by the likelihood ratio test.

## Discussion

The **K **matrix proposed in this study is directly calculated by a simple formula; thus, calculating the inverse of a large matrix can be avoided as in standard methods [5,1,2]. Using NRM with GIAs results in adding more animal records to a dataset for variance component estimation and in calculating genetic evaluation by the mixed model procedure.

The simulation study showed that the estimated genetic variance is reasonably accurate with a high (1-x); however, a low (1-x) resulted in overestimation of the genetic variance, which is caused by false genetic relationships between pairs of GIAs. For instance, in the case of (1-x) equal to 0.0, where the GIAs were erroneously treated as full sibs, a large genetic variance and, consequently, a small residual variance were estimated because the variance component within full sibs was far smaller than that expected for full sibs and vice versa for the variance component between full sibs.

This simulation study assumed perfect genetic relationships between pairs of GIAs. Then, a (1-x) value higher than 0.95 can result in unbiased estimates of additive genetic variance for the simulated data sets. Therefore, a (1-x) value of 0.95 is adequate for the data set of this simulation; however, the choice of the x value may depend on the size of the data set and the genetic constituents of a population: a higher (1-x) may be adequate for a large data set and/or a data set containing monozygotic twins.

In the analysis of real records, a lower (1-x) value is expected because perfect genetic relationships between pairs of GIAs are no longer attainable. The highest genetic relationship is found among monozygotic twins; however, diversity within pairs of twins was found to be larger than expected, according to the human study by Bruder *et al*. [11]. A similar diversity is observed in embryo splitting studies, but manipulating embryos may constitute a potential source of increased diversity. Clones obtained from embryotic cell nuclear transfer may show a higher diversity caused by the recipient cytoplasm [9]. In the case of clones obtained from somatic cell nuclear transfer, an additional source for genetic diversity can originate from mutations in the somatic cell and gene imprinting in the nucleus of the somatic cell [8,10]. Different types of GIAs with various degrees of genetic diversity do exist. Thus, GIAs can be regarded as highly related animals rather than identical animals. Although, (1-x) values can range from 0.0 to 1.0, it is probably nearer to 1.0. To resolve this question, statistical studies such as REML on the estimation of x are needed with a large data set including various types of GIA. The methodology presented here provides an analytical tool to analyse GIAs with an imperfect genetic relationship within pairs of GIAs.

## Competing interests

The authors declare that they have no competing interests.

## Authors' contributions

TO provided the idea of this study, prepared the research plan, initiated the statistical analysis and revised the draft manuscript. KY derived the equation, conducted the simulation study and wrote the draft of this manuscript.
